# Selection for oligotrophy among bacteria inhabiting host microbiomes

**DOI:** 10.1128/mbio.01415-23

**Published:** 2023-08-30

**Authors:** Sara L. Jackrel, Jeffrey D. White, Elisabet Perez-Coronel, Ryan Y. Koch

**Affiliations:** 1 Department of Ecology, Behavior and Evolution, University of California San Diego, La Jolla, California, USA; 2 Department of Biology, Framingham State University, Framingham, Massachusetts, USA; University of Pittsburgh School of Medicine, Pittsburgh, Pennsylvania, USA

**Keywords:** adaptation, genome evolution, host–microbiome, cyanobacterial harmful algal bloom, nutrient limitation, heterotrophic bacteria

## Abstract

Host microbiomes are important regulators of organismal fitness, physiology, and ecology. Microbiomes promote the fitness of their host in part by buffering the host from the full effects of fluctuating conditions and stressors imposed by the external environment. Whether the host conversely serves as a buffer for their associated microbes against variation in the external environment is less clear. Here, we test if bacteria inhabiting the microbiome of a host are locally adapted to nutrient levels in their surrounding external environment. We used a system in which the host, the phytoplankter *Microcystis aeruginosa*, has strains that are locally adapted to low-nutrient versus high-nutrient lakes. Assessing 40 metagenome-assembled genomes belonging to four taxonomic groups of heterotrophic bacteria residing within the host microbiome, we found consistent phylogenetic divergence between strains originating from low-nutrient versus high-nutrient environments. Bacteria found in association with low-nutrient host genotypes obtained from low-nutrient lakes demonstrated genome streamlining, including reduced genome size and fewer sigma factors. These bacterial genomes have features that would facilitate survival in low-nutrient lakes, including (i) greater number of alkaline phosphatase genes that are essential for phosphorus acquisition and (ii) positive selection within genes involved in phosphorus metabolism. Overall, our results demonstrate that despite living in close association with host organisms, bacteria residing within microbiomes may have evolved and undergone environmental selection to stressors external to their host, demonstrating similar patterns of adaptation to those that might be expected to develop among free-living bacteria.

**IMPORTANCE:**

Understanding how natural selection has historically shaped the traits of microbial populations comprising host microbiomes would help predict how the functions of these microbes may continue to evolve over space and time. Numerous host-associated microbes have been found to adapt to their host, sometimes becoming obligate symbionts, whereas free-living microbes are best known to adapt to their surrounding environment. Our study assessed the selective pressures of both the host environment and the surrounding external environment in shaping the functional potential of host-associated bacteria. Despite residing within the resource-rich microbiome of their hosts, we demonstrate that host-associated heterotrophic bacteria show evidence of trait selection that matches the nutrient availability of their broader surrounding environment. These findings illustrate the complex mix of selective pressures that likely shape the present-day function of bacteria found inhabiting host microbiomes. Our study lends insight into the shifts in function that may occur as environments fluctuate over time.

## INTRODUCTION

Recent discoveries have revealed the significance of the host-associated microbiome as a key regulator of host fitness, physiology, and health (1 – 5), with cascading implications for host ecology and regulation of ecosystem function (6 – 9). In particular, a number of studies have demonstrated that microbiomes promote host fitness by buffering the host against the full effects of fluctuating environmental conditions, thus mitigating the effects of environmental stressors on the host (10, 11). However, there has been less study of the converse effect, in which a host buffers its associated microbes against variation in the external environment. Advancing our understanding of the selective pressures shaping bacterial populations that inhabit the microbiome is needed to predict how microbiome community composition and the function of host-associated microbes may fluctuate over space and time. Therefore, we aim to elucidate the role of the host in buffering variation in the external environment among bacteria inhabiting the host microbiome.

Host-associated microbes vary considerably in lifestyle—from those associated with the external surfaces of their hosts to obligate endosymbionts—so, it is useful to consider how selective pressures act on bacteria with lifestyles at the extremes of the free-living versus host-associated gradient. Free-living microbes are directly exposed to fluctuations in environmental conditions, including variation in temperature, pH, salinity, and the concentration of bioavailable nutrients. Such free-living microbes inhabit soils, sediments, air and the water columns of marine and freshwater ecosystems where these taxa are critical regulators of biogeochemical cycles (12, 13). Prior studies have documented a remarkable capacity of free-living microbes to adapt to conditions of their surrounding environment, such as extreme resource efficiency among *Prochlorococcus* inhabiting the low-nutrient open ocean (14). At the opposite extreme of the free-living versus host-associated gradient are obligate endosymbionts that have evolved reduced genomic complexity and are fully dependent on the resources supplied by their host (15). Such obligate endosymbionts are vertically transmitted over host generations, with minimal exposure to the external environment.

We focus instead on host-associated microbes intermediate to these two extremes of free-living versus obligate endosymbionts. Such host-associated microbes experience their surrounding environment through a buffering by their host, often inhabiting microenvironments that are more stable and have relatively benign conditions relative to the stressors of the free-living external environment (16). These host-associated microbes may inhabit the surfaces of animals, the root rhizosphere and leaf phyllosphere of terrestrial plants, and the phycosphere of eukaryotic and cyanobacterial phytoplankton. Such host-associated microbes typically become reliant on exudates, such as sugars and other byproducts of photosynthesis, from a phototrophic host. These host-associated microbes inhabiting the external surfaces of their host and horizontally transmitted symbionts may also move more readily than obligate endosymbionts between the host and the external environment (17). Selective pressures from the external environment have the potential to influence host–microbe interactions, as demonstrated in experimental evolution studies with *Vibrio fischeri* that inhabit both the open ocean and the light organ of their squid host. In this system, adaptation to harsh pH and temperature conditions was shown to significantly alter host–microbe symbioses (18, 19). Understanding whether such adaptation to the external environment is pervasive among host-associated microbiomes in a natural setting could clarify the importance of the external environment in shaping host–microbiome interactions.

Few studies have directly evaluated whether host-associated microbes retain signatures of selective pressure from their external environment, much like-free living microbes. Elucidating this potential role of the external environment would advance our understanding of host–microbiome interactions by building upon prior studies that have demonstrated the capacity for host-associated microbes to evolve to the microenvironment created by their host, as well as for microbiomes to buffer environmental fluctuations for the host and thus reduce the pressure for host evolutionary adaptations (20). Considering the recent increase in awareness of the critical role that host-associated microbes play in regulating host behavior, physiology, fitness, and ecology, it is essential to understand the breadth of pressures that can drive the population dynamics of host-associated microbes. Here, we evaluate whether microbes inhabiting the host microbiome are shaped more so by the resource-rich microenvironment of the host microbiome or a resource-poor external environment.

We investigate whether the phosphorus concentration of freshwater lakes is a selective pressure driving the genomic makeup of heterotrophic bacterial populations that reside within the microbiome of the harmful, bloom-forming cyanobacterium, *Microcystis aeruginosa*. This cyanobacterium forms clonal colonies containing as many as 10^5^ individual cells (21). Embedded within the mucosal polysaccharide matrix that binds these host cells together is a diverse community of heterotrophic bacteria that compose the phytoplankton microbiome or phycosphere (16). We had previously collected colonies of *M. aeruginosa*, which were repeatedly washed to reduce free-living and loosely associated microbes and retain the most closely associated microbes. These colonies were collected from 14 freshwater lakes that vary over 20-fold in phosphorus levels, which is the primary limiting nutrient in freshwater ecosystems (22).

Previous analysis of *M. aeruginosa* metagenome-assembled genomes (MAGs) across this phosphorus gradient revealed clear adaptation of the hosts to their corresponding environment, with strains inhabiting low-nutrient lakes demonstrating greater resource-use efficiency and increased capacity for acquiring phosphorus relative to strains inhabiting high-nutrient lakes. We also previously documented strains that were more closely related to those from low-nutrient lakes, yet residing within high-nutrient lakes, potentially taking advantage of low-nutrient microenvironments within high-nutrient lakes (22). Such low-nutrient microenvironments within high-nutrient lakes are known to develop in lakes experiencing phytoplankton blooms. As these blooms persist for multiple months, the phytoplankton deplete bioavailable nutrients in the water column to low levels (23, 24), thereby providing an available niche for low-nutrient-adapted genotypes of *M. aeruginosa* and their associated heterotrophic bacteria. We previously described three genotypes of *M. aeruginosa,* namely, low-nutrient lake/low-nutrient genotype (LL/LG), high-nutrient lake/high-nutrient genotype (HL/HG), and high-nutrient lake/low-nutrient genotype (HL/LG). We continue the use of this terminology for consistency with our prior work (22), where we found that the heterotrophic bacteria within this microbiome demonstrated metabolic interdependence with their host. Specifically, metagenomic analysis indicated that hosts are reliant on their ubiquitously associated *Aquidulcibacter* spp. to biosynthesize the amino acids threonine, serine, and asparagine, whereas *Aquidulcibacter* spp. derives a significant source of energy from galactose, the primary component of the polysaccharide matrix that binds together colonies of *M. aeruginosa* [note that *Aquidulcibacter* spp. was referred to as *Phycosocius bacilliformis* in Jackrel et al. (22)]. These heterotrophic symbionts, including *Aquidulcibacter* spp., typically remain in association with *M. aeruginosa* in batch cultures maintained in the laboratory (22).

Here, we use this system as a model to investigate whether heterotrophic bacteria inhabiting host microbiomes show evolutionary signatures of adaptation to their surrounding environment. Considering that phytoplankton recruit bacteria into their microbiome from their surrounding environment, there may remain signatures of selection among host-associated bacteria to these external environmental pressures. We assessed 40 metagenome-assembled genomes of four taxonomic groups of heterotrophs found within the microbiomes of three genotypes of *M. aeruginosa* hosts (the low-nutrient LL/LG, the intermediate HL/LG, and the classic high-nutrient HL/HG). We surveyed these heterotrophic MAGs for indicators of genome streamlining that facilitate survival under resource limitation. For example, reduced genome size, low GC content of nitrogenous bases, and increased percentage of coding DNA relative to noncoding nucleotides each facilitate a lower allocation of resources during DNA replication and population growth. Similarly, a reduction in transcription factors or sigma factors indicates reduced complexity and is often associated with oligotrophy (25). Lastly, we surveyed genomes across the phosphorus gradient for variation in gene number and signatures of positive selection in genes associated with phosphorus acquisition and metabolism, such as alkaline phosphatases (26).

## MATERIALS AND METHODS

### Isolate collection

Colonies of *M. aeruginosa* were collected as described in Jackrel et al. (22). In brief, colonies were collected from 14 freshwater lakes in southern Michigan, USA, during July–August 2011 and August 2013. Lakes spanned a wide gradient of ~8–200 µg/L in total phosphorus (TP) concentration, which is a widely used index of lake productivity. This range of TP encompasses that of over 82% of lakes in the Northeastern USA (27). Water was collected from the mixed layer of each lake via a 12-m long integrated tube sampler, and subsets were stored for the measurement of lake TP via the molybdenum-blue colorimetric method with a persulfate digestion (28, 29). Standard thresholds for TP were used to assign lake trophic state, including 10 µg/L and 30 µg/L for the oligotrophic–mesotrophic and mesotrophic–eutrophic boundaries, respectively (30). TP measurements were taken for each lake at least three times during multiple years with the exception of Lake Lansing, which was sampled twice. Longer-term data sets for the nutrient content of these lakes can be found in Jackrel et al. (22).

Individual colonies of *M. aeruginosa* were isolated using a Pasteur micropipette and dissecting scope. While some large *M. aeruginosa* colonies are amorphous, loose aggregations of cells, we selected only smaller compact colonies that were distinctive in shape. To remove free-living bacteria and retain only closely associated bacteria within the host microbiome, including those embedded in the intercellular mucilage of the colony, we washed individual colonies by sequentially pipetting through a series of six-well plates containing sterile 0.5× WC-S growth medium. Similar washing steps have been proven effective at removing free-living microbes, and the use of WC-S growth medium should disfavor the survival of accompanying free-living heterotrophic microbes in the absence of an organic carbon source (31 – 33). Washed colonies were initially inoculated into 20 mL tubes of sterile WC-S growth medium and then maintained in 200 mL batch cultures in 0.5× WC-S growth medium at 23°C under a 12:12 h light:dark cycle of 80 µmol m^−2^ s^−1^.

### Amplicon and metagenomic sequencing

Biomass of each culture was collected, and DNA was extracted and sequenced as described in Jackrel et al. (22). In brief, samples of each *M. aeruginosa* culture were concentrated on 0.45 µm nitrocellulose filters, frozen immediately, and stored at −80°C. DNA was extracted using a Qiagen DNeasy Blood and Tissue Kit. The host−microbiome community of bacteria was surveyed by sequencing PCR amplicon of the V4 region of the 16S rRNA gene with 515f/806c primers on a 2 × 250 Illumina MiSeq v2 run at the University of Michigan Medical School. Metagenomic libraries were generated with a 500-nt insert size using a Warfergen Biosystems Apollo 324 library preparation system. Metagenome sequences of the host and associated bacteria were generated on an Illumina HiSeq 100 cycle 2 × 100 nt PE run at the University of Michigan Sequencing Core. All raw sequencing data files are publicly accessible under SRA PRJNA351875. We trimmed raw metagenomic reads of adapters using Scythe and quality-trimmed reads using Sickle with default parameters (34). We assessed sequence quality before and after quality filtering using FastQC. We assembled sequencing reads into contigs using idba-ud with the following parameters: --mink 50, --maxk 92, --step 4 or 6, and --min_contig 500 (35). We then imported sequences of a minimum of 2 kb into VizBin, which uses nonlinear dimension reduction of tetranucleotide genomic signatures to bin contigs into taxonomic groupings. We manually selected and extracted clusters of sequences as metagenome-assembled genome bins. We partitioned sequences within each bin into sample-specific FASTA files to generate MAGs. We determined percentage completeness, GC content, genome size, and coding density of each MAG using checkM (36). We summed sigma factors within each MAG by counting the number of genes assigned to any of the 27 protein families containing the keyword “sigma” within either the protein family name or protein family summary. To test whether these streamlining metrics among heterotrophs differed across our three host groups, we used linear mixed-effects models using the lmer function in the lme4 package in R. For each model, one of the streamlining metrics was the dependent variable, host genotype was the fixed effect, and taxonomic grouping of the MAG was the random effect. We report the marginal *R* ^2^ value for each model to describe the variance explained by the fixed factor (i.e., host genotype) (37). Due to our prior results on genome evolution in *M. aeruginosa* as well as predictions from genome streamlining theory, we then applied our a priori ordered predictions to calculate corrected *P*-values with a directional analysis of variance test using Spearman’s rank correlations (38). We also used phylogenetic comparative methods to test for a phylogenetic signal within each streamlining metric of heterotrophs across host groups. Specifically, we computed a phylogenetic analysis of variance with a directional correction test for our a priori ordered predictions. We also calculated Pagel’s *λ* and Moran’s *I* to probe for a phylogenetic signal using the R package phytools (39 – 41). For these phylogenetic approaches, we constructed a phylogeny of all four taxonomic groups built using the *gyrB* housekeeping gene with RAxML (42).

We focused our analysis on four taxonomic groups that each contained representative MAGs from at least two of the three genotypes of the *M. aeruginosa* host. Within these groups, we retained all high- and medium-quality draft genomes as defined by Bowers et al. (43), which requires a minimum of 50% completeness and less than 10% contamination (43). We uploaded all high-quality draft MAGs to JGI Gold, and all high-quality and medium-quality draft MAGs into KBase. We determined the taxonomic identity and the pairwise average nucleotide identity (ANI) using the GTDB-Tk Classify and FastANI functions in KBase, respectively. After eliminating outlier MAGs as determined based on divergent taxonomic assignments and/or ANIs, we were able to extract a total of 16 MAGs of *Aquidulcibacter* spp. (family *Hyphomonadaceae*) that were a minimum of 70% complete with under 5% contamination, 7 MAGs of *ELB16-189* spp. (family *Cyclobacteriaceae*) that were a minimum of 54% complete with under 9% contamination, 10 MAGs of *SM1A02* spp. (family *Phycisphaeraceae*) that were a minimum of 73% complete with under 4% contamination, and 7 MAGs in the family *Burkholderiacea* that were a minimum of 53% complete with under 4% contamination (see full statistics in Table S1). We did not dereplicate our MAGs because we aimed to detect genomic differences derived from independently collected samples from across a phosphorus gradient (44). We constructed multilocus sequence typing phylogenies for each of the four taxonomic groups of heterotrophic bacteria. We used representative gene sequences of five housekeeping genes (*pgi*, *gltX*, *ftsZ*, *glnA*, and *gyrB*) from each taxonomic group to search for gene orthologs in the metagenomic data of each MAG by making custom blast databases and using the blastdbcmd command to extract sequence ranges based on blast output coordinates. For blast searches that failed to yield a gene match, we extracted gene sequences from JGI Gold annotations for each MAG, when available. We concatenated extracted gene sequences, aligned sequences with MUSCLE using default parameters, and trimmed alignments with Geneious (45). We constructed phylogenies using RAxML with *Caulobacter vibrioides*, *Bacteroidetes cellulosilyticus*, *Bacillus subtilis,* and *Burkholderia psuedomallei* used as outgroups of *Aquidulcibacter* spp., *ELB16-189* spp*.*, *SM1A02* spp*.*, and *Burkholderiacea*, respectively (42). Outgroup gene sequences were obtained from NCBI. Newick phylogenies were visualized in ggTree (46).

We then annotated these 40 MAGs using the KBase Annotate Microbial Assembly with RASTtk-v1.073 tool to generate protein family frequency tables. We used these tables to first complete an untargeted analysis to determine whether any protein families within these MAGs were associated with different phylogenetic groups of their *M. aeruginosa* host. We ran separate analysis of variance models with false discovery rate corrections for each of the four taxonomic groupings of heterotrophic bacteria using STAMP (47). We then completed a targeted analysis of genes known to be important in phosphorus metabolism. For this analysis, we identified all relevant protein families by searching family descriptions at http://pfam.xfam.org/families and JGI Gold annotated MAGs for the following keywords: “alkaline,” “phosphatase,” “Pho,” “phosphorus,” “phosphonate,” and “Phn.” Since we had a priori expectations based on a prior study of the *M. aeruginosa* host across this phosphorus gradient, we used directional analysis of variance for this analysis (38). Lastly, we tested for evidence of positive selection within all genes found in the low-nutrient branches of each taxonomic group of heterotrophic bacteria. We computed synonymous-to-nonsynonymous substitution rate ratios using default parameters in the PosiGene software package (48). Analyses were completed separately for each taxonomic group of heterotrophic bacteria, with orthologs identified against the most complete genome within each family as the anchor species (i.e., *Aquidulcibacter* K13-06, *ELB16-189* L211-101, *Burkholderiaceae* BS13-02, and *SM1A02* K13-06). To account for multiple comparisons, we applied a false-discovery rate correction.

## RESULTS

**Fig 1:**
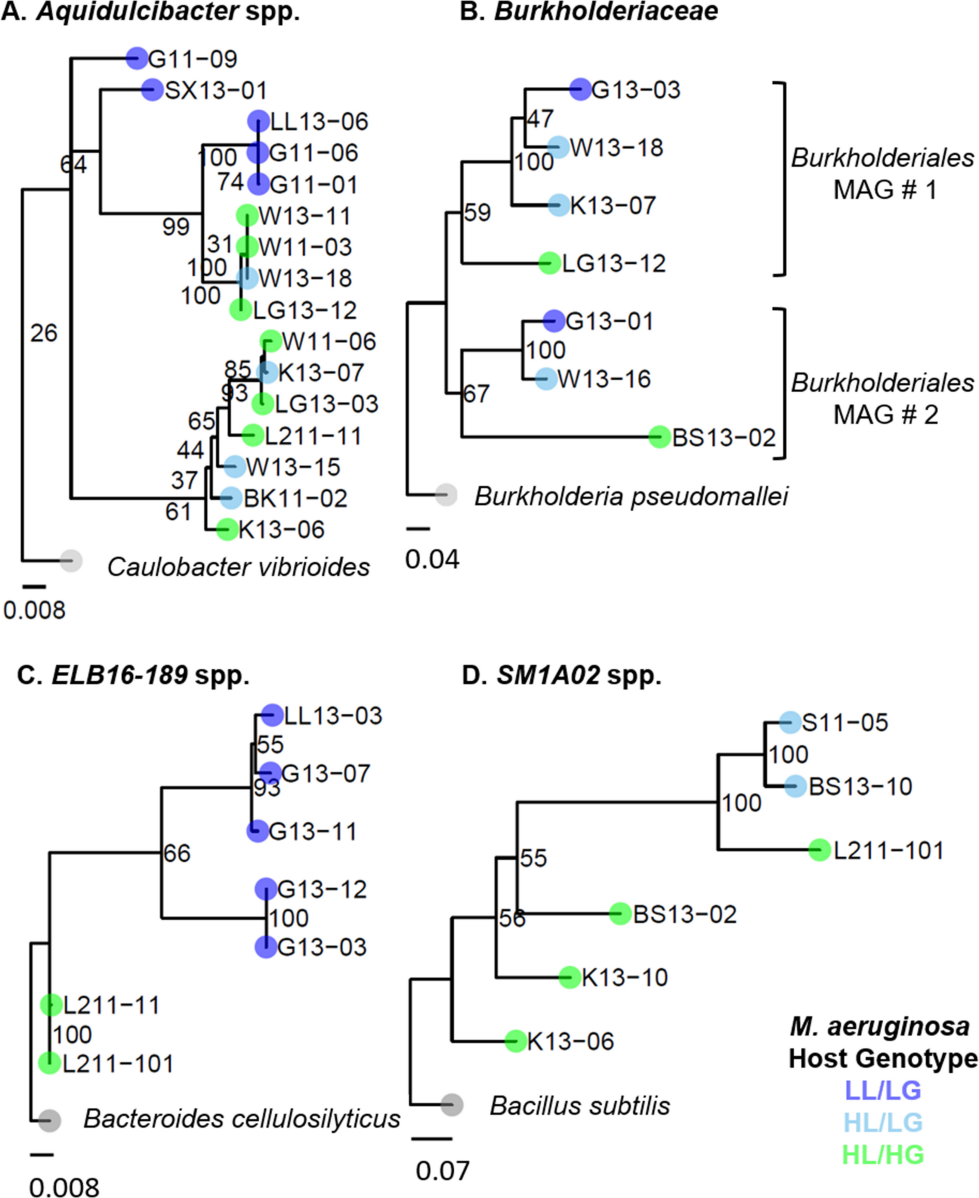
Divergent genome structure across phylogenies of four taxonomic families of heterotrophic bacteria associated with their host *Microcystis aeruginosa* collected across a phosphorus gradient of inland lakes in Michigan, USA. Multilocus sequence typing was used to determine evolutionary history with RAxML based on concatenated housekeeping genes (including ftsZ, glnA, gltX, gyrB, and pgi). Shown are the four heterotrophic groups with representatives from across the phosphorus gradient: (A) *Aquidulcibacter* spp., (B) *Burkholderiaceae*, (C) *ELB16-189* spp., and (D) *SMA102* spp. Green circles indicate heterotrophs associated with a HL/HG host, light blue indicates those associated with a HL/LG host, and dark blue indicates those associated with LL/LG hosts. Strain names include an abbreviation for the lake and year (i.e., G11-09 is the ninth colony collected from Gull Lake in 2011). Note *Burkholderiaceae* MAGs were found in two separate metagenome bins, with a gradient evident within each bin. Additionally, *Phycisphaerales* MAGs, including *SM1A02* spp., were found in multiple metagenome bins; so, shown above are results from the metagenome bin that contained the largest number of MAGs. Phylogenetic distance of the outgroups is reduced by factors of 10 or 100× to aid in visualization.

**Fig 2:**
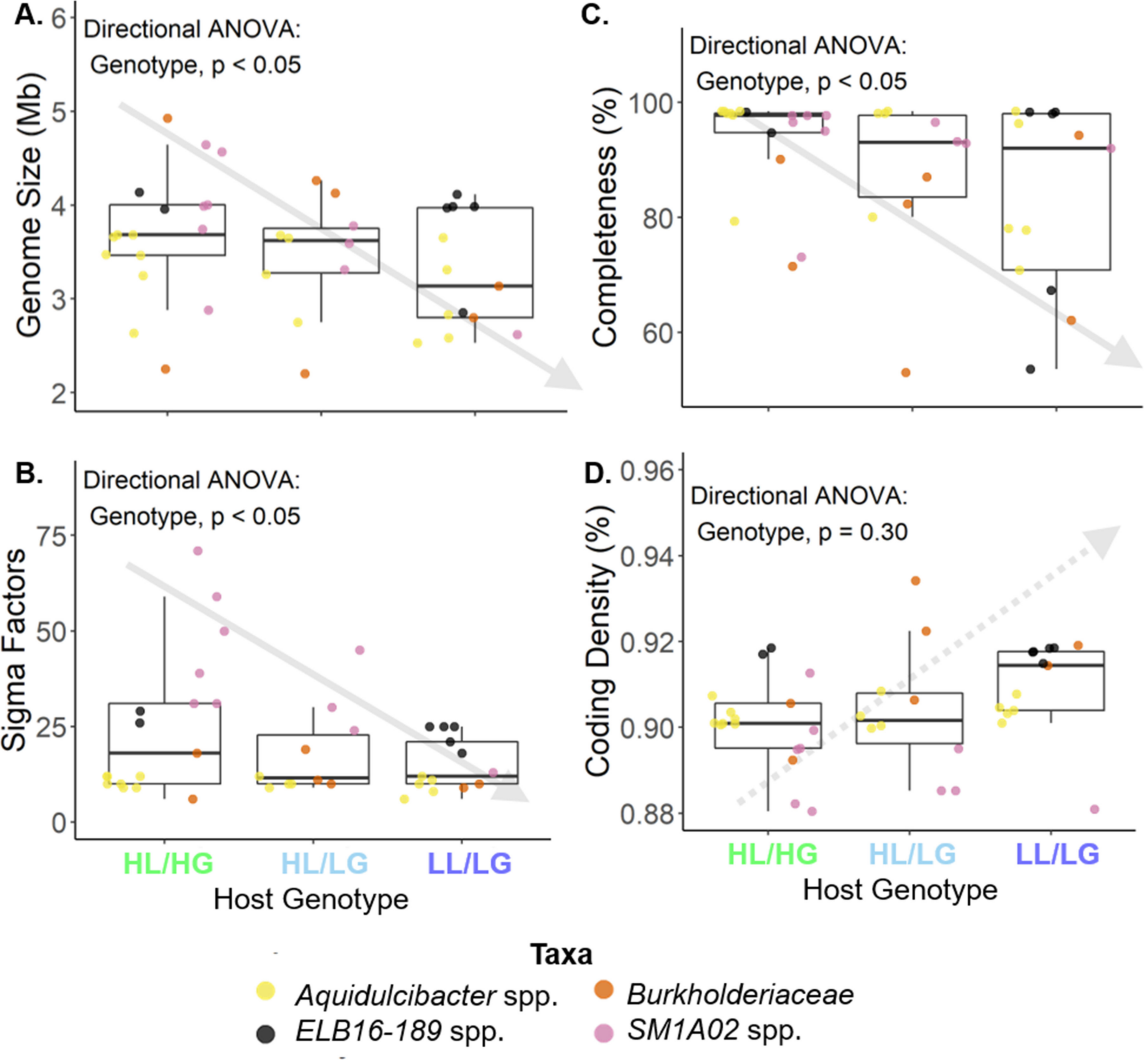
Heterotrophic bacteria found in close association with their *Microcystis aeruginosa* hosts demonstrate genome streamlining and reduced organismal complexity when residing in low-nutrient environments. When associated with *M. aeruginosa* collected from low-nutrient lakes (LL/LG) and those collected from high-nutrient lakes but with low-nutrient-type genomes (HL/LG), heterotrophic bacteria tended to have (A) reduced genome size (Mb), (B) fewer sigma factors, and (C) genomes with fewer core genes compared to those associated with high-nutrient HL/HG hosts. (D) Heterotrophic bacteria associated with LL/LG hosts also tended to have a higher percentage coding density; however, this trend was not statistically significant. Linear mixed-effects models with a directional correction test for our a priori ordered predictions within each of the four taxonomic groups by setting taxonomic group as a random factor were used. Trends should therefore be broad across taxa and not driven by any single taxonomic group.

**Fig 3:**
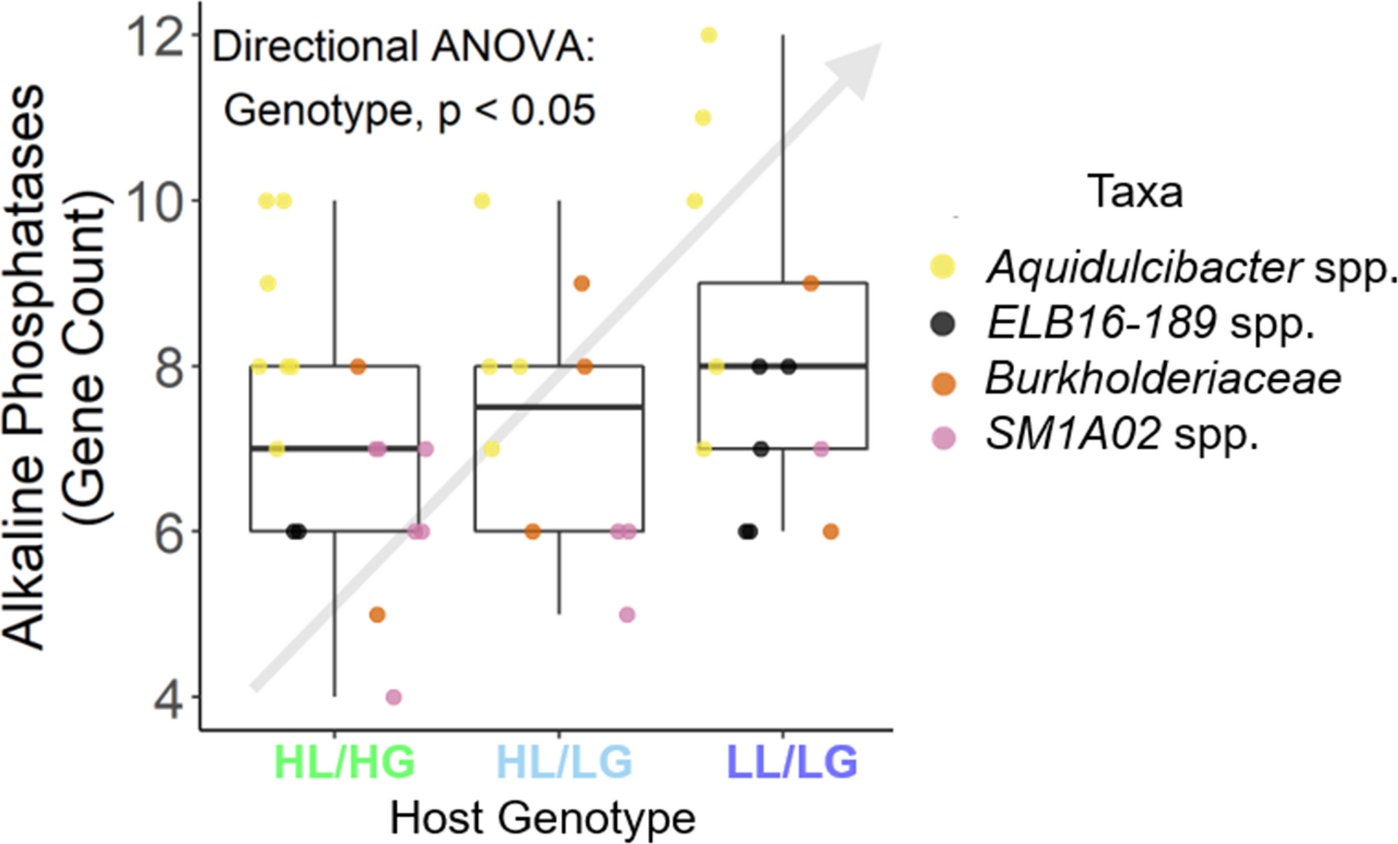
Heterotrophic bacteria found in close association with their *Microcystis aeruginosa* hosts demonstrate directional selection for an increased ability to acquire phosphorus when residing in low-nutrient environments. Heterotrophs associated with the most low-nutrient environments (i.e., associated with LL/LG hosts from low-nutrient lakes) contained the highest number of alkaline phosphatases. Linear mixed-effects models with a directional correction test for our a priori ordered predictions within each of the four taxonomic groups by setting taxonomic group as a random factor were used. Trends should therefore be broad across taxa and not driven by any single taxonomic group.

Our data set included 40 high- and medium-quality draft MAGs of heterotrophic bacteria belonging to four taxonomic families that were obtained from enrichment cultures of 28 strains of *M. aeruginosa*. These families include *Cyclobacteriaceae*, *Burkholderiaceae*, *Hyphomonadaceae*, and *Phycisphaeraceae*. Detailed quality information for each MAG is provided in Table S1. Additional MAGs belonging to other taxonomic groups were found only in association with one genotype of *M. aeruginosa* (i.e., LL/LG hosts or HL/HG hosts) and were excluded because these incomplete groups did not permit comparison of representative MAGs from across our phosphorus-based gradient of freshwater lakes. We found each of these four taxonomic groups diverged across the phosphorus gradient based on concatenated housekeeping genes (including *ftsZ*, *glnA*, *gltX*, *gyrB*, and *pgi*) (Fig. 1). These patterns of divergence across the phosphorus gradient are also evident by comparing pairwise average nucleotide identities across whole MAGs (see Table S2). We found that the genomes of heterotrophic bacteria found in association with LL/LG *M. aeruginosa* hosts collected from low-nutrient lakes demonstrated multiple indicators of evolutionary adaptation of oligotrophy. When assaying genome-wide trends of these heterotrophic bacteria, our results match the expectations of genome streamlining theory across a phosphorus-based gradient of freshwater lakes. Specifically, bacteria associated with LL/LG *M. aeruginosa* hosts tended to have reduced genome size, fewer sigma factors, and fewer core genes within their genomes compared to bacteria associated with the HL/HG *M. aeruginosa* hosts (Fig. 2A through C, linear mixed-effects models with directional analysis of variance, *n* = 40 MAGs; Fig. 2A Genome Size: host genotype fixed effect *P* < 0.05, marginal *R* ^2^ = 0.098; Fig. 2B Sigma Factors: host genotype fixed effect *P* < 0.05, marginal *R* ^2^ = 0.044; Fig. 2C % Completeness: host genotype fixed effect *P* < 0.05, marginal *R* ^2^ = 0.078). Similarly, bacteria associated with LL/LG hosts tended to have a higher percentage of coding density as predicted by genome streamlining theory; however, this trend was not statistically significant (Fig. 2D). Furthermore, we found that these bacteria demonstrated directional selection for a greater number of alkaline phosphatases (Fig. 3). Phylogenetic comparative methods also indicated significant trends for each of these metrics (Fig. 2A Genome Size: phylogenetic ANOVA *P* < 0.10, Pagel’s *λ* = 0.924, *P* = 0.031, Moran’s *I* = 0.112, *P* = 0.088; Fig. 2B Sigma Factors: phylogenetic ANOVA *P* < 0.05, Pagel’s *λ* = 0.998, *P* < 0.001, Moran’s *I* = 0.661, *P* = 0.001; Fig. 2C Completeness: phylogenetic ANOVA *P* < 0.05, Pagel’s *λ* = 0.316, *P* = 0.21, Moran’s *I* = 0.152, *P* = 0.054; Fig. 2D Coding density: phylogenetic ANOVA *P* < 0.05, Pagel’s *λ* = 0.994, *P* < 0.001, Moran’s *I* = 0.560, *P* = 0.001; Fig. 3 Alkaline phosphatases: phylogenetic ANOVA *P* < 0.10, Pagel’s *λ* = 0.344, *P* = 0.0012, Moran’s *I* = 0.408, *P* = 0.001).

In addition to alkaline phosphatases, we also more broadly surveyed all genes involved in phosphorus metabolism. We found that within each of the four taxonomic groups of heterotrophic bacteria, there was evidence of positive selection acting on genes involved in phosphorus metabolism among the low-nutrient phylogenetic branch (Table 1). Enzymatic genes under positive selection among bacteria associated with LL/LG *M. aeruginosa* included those for glycerophosphodiester phosphodiesterase and glycerol-3-phosphate dehydrogenase for *Aquidulcibacter* spp.; carbamoyl-phosphate synthase for *Burkholderiaceae*; phosphoglycerate kinase, (d)CMP kinase, and *N*-acetyl-gamma-glutamyl-phosphate reductase for *ELB16-189* spp.; and glutamine-fructose-6-phosphate transaminase and nucleoside diphosphate kinase for *SM1A02* spp. Additional genes under positive selection are described in Table S3. In addition to our targeted analysis of divergence across the phosphorus gradient in alkaline phosphatases, we also conducted an untargeted analysis of gene differences across the phosphorus gradient by surveying all genes identified within MAGs. We report all 76, 54, and 21 genes that significantly differed across the phosphorus gradient in *ELB16-189* spp., *Burkholderiaceae*, and *Aquidulcibacter* spp., respectively, in Fig. S1 through S3. Some notable findings include a phosphohydrolase domain (PF13286) that occurred only within LL/LG-associated *ELB16-189* spp., whereas a phosphate starvation gene (PF06146) and nitrogen regulatory protein (PF00543) occurred only in HL/HG-associated *ELB16-189* spp. Within *Aquidulcibacter* spp., we found a calcineurin-like phosphoesterase (PF12850) and bacterial flagellin (PF00669) each in greater abundance among LL/LG than HL/HG-associated strains, whereas bacterial regulatory proteins (PF0044) were found in greater abundance among HL/HG strains.

**TABLE 1:** Genes under positive selection for the low-nutrient branches of heterotrophic bacteria associated with their *Microcystis aeruginosa* host across a phosphorus gradient of freshwater lakes in Michigan^*a*^

Taxon	Gene function	GenBank accession no.	W	FDR *P*-value
*Aquidulcibacter* spp.	Glycerophosphodiester phosphodiesterase	OYU75423.1	19.5	<0.0001
*Aquidulcibacter* spp.	Glycero-3-phosphate dehydrogenase	OYU75702.1	6.9	0.0093
*Burkholderiaceae*	Carbamoyl-phosphate snythase large subunit	MBL8534549.1	17.9	0.015
*Burkholderiaceae*	Adenylate kinase	MBK8018599.1	41.0	0.007
*ELB16-189* spp.	Histidine kinase	NOS56772.1	20.2	0.0036
*ELB16-189* spp.	Phosphoglycerate kinase	MCE2893864.1	34.7	0.0069
*ELB16-189* spp.	(d)CMP kinase	MCE2893935.1	10.1	0.0069
*ELB16-189* spp.	*N*-acetyl-gamma-glutamyl-phosphate reductase	NOS54628.1	38.6	0.0073
*SM1A02* spp.	Glutamine-fructose-6-phosphate transaminase	MBX3410265.1	8.1	0.038
*SM1A02* spp.	Nucleoside diphosphate kinase	MCG3122206.1	22.7	0.038

^*a*^ Genes shown are those involved in phosphorus cycling and metabolism. A multiple comparisons correction was applied to significance values using a false-discovery rate (FDR). See Table S2 for all genes under positive selection. All results generated including ω (dN/dS) were computed in the PosiGene software using default parameters.

## DISCUSSION

Our results demonstrate that populations of host-associated microbes have undergone selection by the broader external environment. Four host-associated taxonomic families of heterotrophic bacteria showed adaptation across a phosphorus gradient through genome-wide signatures of streamlining and an increased capacity to both acquire and metabolize phosphorus. These trends apply broadly across all taxonomic families tested, rather than being driven by a single taxonomic group, due to our linear mixed-effects modeling approach. We reached similar biological conclusions of genome streamlining across multiple families of host-associated microbiomes using phylogenetic comparative methods. In combination with our prior results demonstrating metabolic interdependence between *Aquidulcibacter* spp. and their host, these results elucidate that both the host and the environment are selective pressures shaping the bacterial populations inhabiting host microbiomes.

Adaptive responses of these host-associated heterotrophs in low-nutrient environments included metrics of genome streamlining, where resource limitation is expected to select for reduced resource use in DNA replication and overall reduced complexity of the genome. Specifically, we found reduced genome size and fewer sigma factors among heterotrophs inhabiting low-nutrient conditions. A smaller genome requires fewer resources during DNA replication, and fewer sigma factors broadly indicate selective pressure against large, complex genomes. Furthermore, heterotrophs associated with the intermediate host group HL/LG were found to be intermediate relative to those associated with the HL/HG and LL/LG hosts in most metrics. This intermediate pattern was more evident for certain taxonomic groups, particularly *SM1A02* spp. This suggests that these heterotrophs associated with HL/LG may have adapted to survive within low-nutrient microenvironments within high-nutrient lakes. While certain metrics were notably weaker for some taxonomic groups, including the number of sigma factors for *Aquidulcibacter* spp. and coding density for *SM1A02* spp., the consensus from our linear-mixed modeling and phylogenetic comparative approaches indicate that genome streamlining is prevalent throughout *Microcystis*-associated heterotrophic bacteria. Some of these weaker trends may be due to smaller sample sizes and/or incomplete metagenome-assembled genomes. Still, considering the consistency of these results across each of the four heterotrophic taxa surveyed in this study, selection of traits among host-associated bacteria by external environmental conditions may be a wide-scale phenomenon. Indeed, these results are consistent with a recent study that found selection for genome streamlining under stressful environmental conditions among isolates of the free-living *Bradyrhizobium diazoefficiens* bacterium inhabiting the soil at the base of their acacia plant mutualists. We might elucidate the broader rules regulating trait selection among hosts and their associated bacteria through further studies that compare the degree of trait selection across different host–microbe systems experiencing varying types of stress gradients and degrees of host-association versus free-living lifestyles (49, 50).

We found multiple lines of evidence for the adaptation of host-associated microbes to oligotrophy, suggesting the external environment is a major selective force. First, we observed an expansion of alkaline phosphatase genes and positive selection for other genes involved in the efficient acquisition and metabolism of phosphorus, certainly adaptive under phosphorus-limited growth conditions. Second, the genomes of LL/LG-associated *Aquidulcibacter* spp. suggested increased cellular motility due to a greater abundance of bacterial flagella. Although coming at a high energetic cost, increased motility would conceivably facilitate the formation of host–microbe associations in low-nutrient environments that harbor lower densities of hosts (relative to high-nutrient environments). Flagella also enable microbes to mix the diffusive boundary layer surrounding them as nutrients become depleted in their host microenvironment, further enhancing nutrient scavenging ability under lower-phosphorus conditions. Third was the loss of genes related to environmental sensing, including a phosphate starvation gene and nitrogen regulatory gene among LL/LG-associated *ELB16-189* spp. This result aligns with prior surveys of marine bacteria that identified the loss of two-component sensory systems as a hallmark of oligotrophy. These two-component sensory systems are the most common environmental sensing system among bacteria for detecting short-term shifts in nutrient availability, light, and temperature (51).

There is also substantial evidence that each of the four families included in our study forms close associations with *M. aeruginosa* across space and time. For example, *Aquidulcibacter* spp. have been identified in association with *Microcystis* spp. in Asia, North America, Europe, and Africa, with some close relatives of *Aquidulcibacter* spp. found as sessile cells embedded within the extracellular matrix of the algal phycosphere (52 – 54). *ELB16-189* spp. has been found in association with *M. aeruginosa* colonies isolated from Lake Erie (55). Further suggesting a persistent association with its host, the genome of *ELB16-189* spp. contains *mlrA*, which encodes for an enzyme to degrade the microcystin cyanotoxin produced by its host (55, 56). *Burkholderiaceae* have also been found in Asia and North America in association with *M. aeruginosa* and have genomic content indicative of an ability to degrade microcystin (57 – 59). Lastly, *SM1A02* spp. is an anaerobic ammonium oxidizing bacterium found in association with *M. aeruginosa* blooms in Asia and Australia (60, 61). *SM1A02* spp. has also been found in high abundance within the phycosphere of the bloom-forming cyanobacterium *Raphidiopsisraciborskii* as well as marine dinoflagellates (62, 63).

Integrating our current findings with our past work in this system, we found several consistent ways in which low-nutrient stress caused trait selection in both the *M. aeruginosa* host and their associated heterotrophic bacteria. Both the host and their associated bacteria from low-nutrient environments shared indicators of genome streamlining, including percentage of coding DNA, number of sigma factors, and completeness. Both also demonstrated the selection for increased nutrient affinity, including increased gene copy number and/or signatures of positive selection of alkaline phosphatases, histidine kinases, and glutamate synthase. Determining whether this consistency in trait selection resulted from independent selection on hosts and their associated bacteria, or through co-evolutionary interactions, would be valuable for future study. Comparing the responses of hosts and their associated bacteria to different types of stressors may shed light on the relative roles of independent versus co-evolutionary trait selection. For example, in contrast to pressure to acquire phosphorus, a universally required nutrient, other selective pressures may differentially affect the host versus its associated microbes due to differing physiologies. Turbidity, for example, would be expected to be a substantial stressor for a photosynthetic host but have only limited direct effects on associated heterotrophic bacteria. Comparing the patterns of trait selection on paired hosts and their associated bacteria across different types of selective gradients might clarify the underlying mechanisms of evolutionary change in host–microbiome systems.

Results from our study suggest that environmentally mediated selection of host-associated bacteria may have played a role in the recent expansion of blooms of *M. aeruginosa* and other undesirable planktonic cyanobacteria into low-nutrient habitats, where they are not typically expected (64, 65). Reduction of nutrient loading into freshwater environments is one of the most frequently used and historically effective mitigation efforts for controlling harmful phytoplankton blooms. Understanding the adaptation of both the host *M. aeruginosa* and heterotrophs within its microbiome to oligotrophy may guide the development of new mitigation efforts that may prove more effective against harmful phytoplankton. The negative environmental effects of harmful phytoplankton blooms are predicted to intensify with climate warming (66, 67). Therefore, fully understanding the potential for genetic changes and subsequent directional selection within both the host and host microbiome may aid in predicting future range shifts and the mitigation of the negative effects of these blooms on freshwater ecosystem function and human health.

Beyond selective pressures shaping bacterial populations within the host microbiome, we have previously identified ecological shifts in the community composition of the *M. aeruginosa* microbiome across the phosphorus gradient via 16S rRNA amplicon sequencing (22). In particular, *Aquidulcibacter* spp., the most abundant taxon in the *M. aeruginosa* microbiome, comprised the greatest proportion of the microbiome community among HL/HG genotypes and the least among LL/LG genotypes. *Cyclobacteriaceae* and *Burkholderaldes* (the third and sixth most abundant taxa, respectively) showed the opposite trend, comprising a larger proportion of the community among LL/LG genotypes. Considering that we found similar patterns of local adaptation in each of the four heterotrophic taxa studied, the driving factors behind these ecological shifts in community composition remain unclear. Future studies may clarify the drivers of host microbiome shifts across habitats by assessing both ecological and evolutionary processes across resource gradients. Additionally, to understand the potential interactions between host–microbe ecology and microbial evolutionary change, future studies could probe whether the degree of host dependency of a bacterium corresponds with the degree of streamlining found in the bacterium’s genome. Such future work could measure host–microbe interdependency through metagenomic inferences, as we have done previously with *Aquidulcibacter* spp., or more directly through co-culture experiments (22). While we found evidence of genome streamlining among all taxa tested in this study, a systematic analysis of the magnitude of genome streamlining among all bacteria within a host microbiome could investigate whether this evolutionary trajectory corresponds with those bacteria that harbor the closest associations with their host.

It is important to note that our study has limitations inherent in all comparative studies of metagenome-assembled genomes. Apparent gene loss, such as loss of core genes among oligotrophic MAGs, could also result from incomplete MAGs due to the limitations of metagenomic assembly. We aimed to minimize the possibility of incorrect inferences of gene loss by using only high- and medium-quality MAGs as defined by Bowers et al. (43). Furthermore, our results should also be considered within the context of multiple simultaneous drivers of selection acting on members of the host microbiome. As previously demonstrated, trait selection of the host *M. aeruginosa* has led to different ecotypes proliferating depending on lake nutrient status. It is conceivable that these evolutionary changes within *M. aeruginosa* could influence the composition and concentration of exudates within the phytoplankton phycosphere. Host genotype has been found to alter the composition of exudates and nutrients within plant rhizospheres (68). Therefore, without further study of precisely how the composition of exudates and nutrients in the microbiome varies among *M. aeruginosa* genotypes, we cannot disentangle whether the evolutionary changes we have observed among heterotrophs are a result of direct selection from the external environment or an indirect effect of the external environment on the host. Regardless of whether the evolutionary changes that we observed within this system are a result of direct or indirect effects, our results highlight the important role of the external environment in ultimately driving evolutionary change among host-associated microbes.

Our work highlights the need for future studies aimed at disentangling the relative roles of the host versus the external environment in driving the evolutionary trajectories of host-associated microbes. Controlled experimental evolution studies could be employed to quantify evolutionary changes within host-associated microbes in response to shifts in host identity versus shifts in external environmental conditions. Considering the recent accumulation of evidence that host-associated microbes regulate many aspects of animal and plant host physiology and ecology, quantifying the role of external environmental stressors in driving microbial evolution of host-associated taxa may aid predictions of how host fitness and ecology may shift under changing environmental conditions.

## SUPPLEMENTAL MATERIAL

Supplemental figures and tables mbio.01415-23-s0001.docx Tables S1 to S3; Fig. S1 to S3.

ASM does not own the copyrights to Supplemental Material that may be linked to, or accessed through, an article. The authors have granted ASM a non-exclusive, world-wide license to publish the Supplemental Material files. Please contact the corresponding author directly for reuse.
