# Supplementary material for: Selection for oligotrophy among bacteria inhabiting host microbiomes
Source: mBio. 2023 Aug 30;14(5):e01415-23. doi: 10.1128/mbio.01415-23 (PMC10653850; doi:10.1128/mbio.01415-23)
Supplement: Supplemental figures and tables — Tables S1 to S3; Fig. S1 to S3. [file mbio.01415-23-s0001.docx]

**Supplementary Materials**

**Table S1.** Assembly quality and collection information for each MAG included in further analyses. Lake from which the *M. aerginosa* host was collected is described, including the mean soluble reactive phosphorus (SRP) for these lakes. Coloring of samples indicates whether the bacterium was associated with a HL/HG host (green), HL/LG host (light blue) or LL/LG host (dark blue). Contamination and completeness information generated from checkM. Bacterial taxonomy reported to genus when possible (phylum = P, class = C, order = O, family = F and genus = G).

**Table S2.** A) Average nucleotide identities (ANI) of 16 MAGs of *Aquidulcibacter* spp. (Phylum: *Proteobacteria*; Class: *Alphaproteobacteria*; Order: *Caulobacterales*; Family: *Hyphomonadaceae*; Genus: *Aquidulcibacter*)*.* B) ANI of 7 MAGs of *Burkholderiaceae* (Pylum: *Proteobacteria*; Class: *Gammaproteobacteria*; Order: *Burkholderiales*; Family: *Burkholderiaceae*). C) ANI of 7 MAGs of *ELB16*-*189* spp. (Phylum: *Bacteroidota*; Class: *Bacteroidia*; Order: *Cytophagales*; Family: *Cyclobacteriaceae*). D) ANI of 10 MAGs of *SM1A02* spp. (Phylum: *Planctomycetota*; Class: *Phycisphaerae*; Order: *Phycisphaerales*: F: *Phycisphaeraceae*). Colors indicate the phylogenetic grouping of the *Microcystis aeruginosa* host colony that each bacterium was found in close association with within the phytoplankton microbiome.

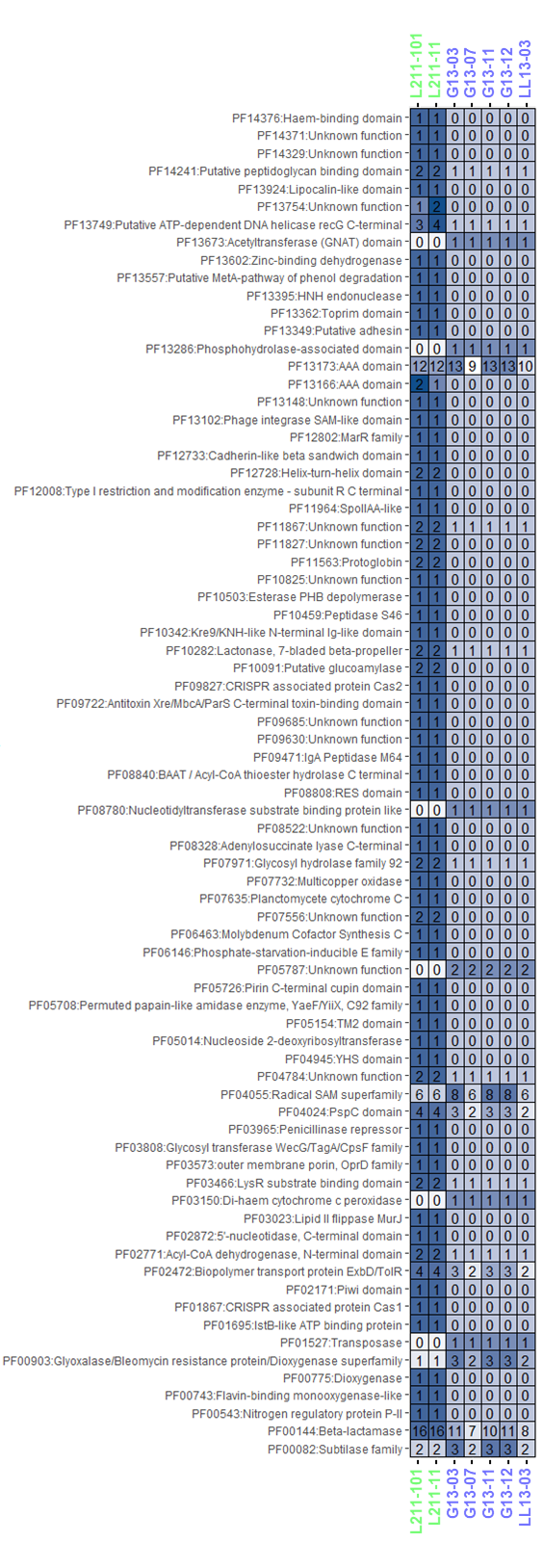


**Figure S1.** Metagenome assembled genomes (MAGs) of *ELB16-189* spp. found in association with their *Microcystis aeruginosa* hosts collected from inland lakes of Michigan differed significantly in the abundances of protein families across two different phylogenetic groups of *M. aeruginosa.* Heatmap color depicts gene counts for each MAG, where lighter colors indicate fewer genes identified in that MAG.


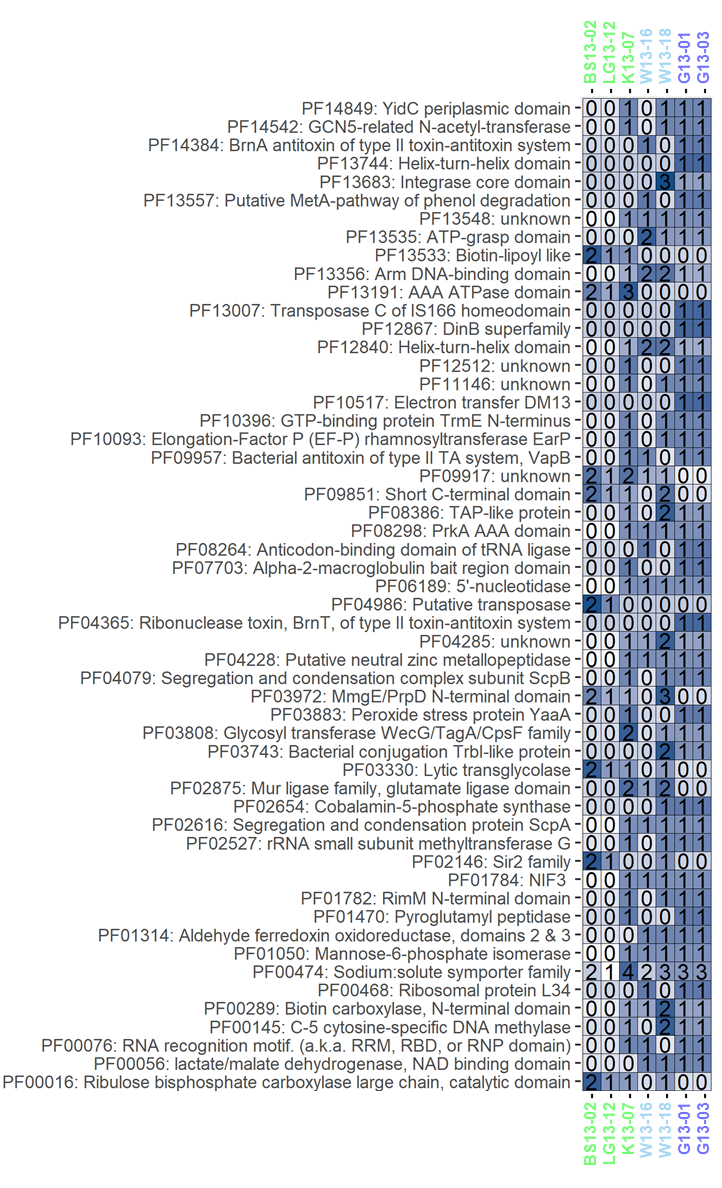


**Figure S2.** Metagenome assembled genomes (MAGs) of *Burkholderiaceae* found in association with their *Microcystis aeruginosa* hosts collected from inland lakes of Michigan differed significantly in the abundances of protein families across two different phylogenetic groups of *M. aeruginosa.* Heatmap color depicts gene counts for each MAG, where lighter colors indicate fewer genes identified in that MAG.

**Figure S3.** Metagenome assembled genomes (MAGs) of *Aquidulcibacter* spp. found in association with their *Microcystis aeruginosa* hosts collected from inland lakes of Michigan differed significantly in the abundances of protein families across three phylogenetic groups of *M. aeruginosa*. Heatmap color depicts gene counts for each MAG, where lighter colors indicate fewer genes identified in that MAG.


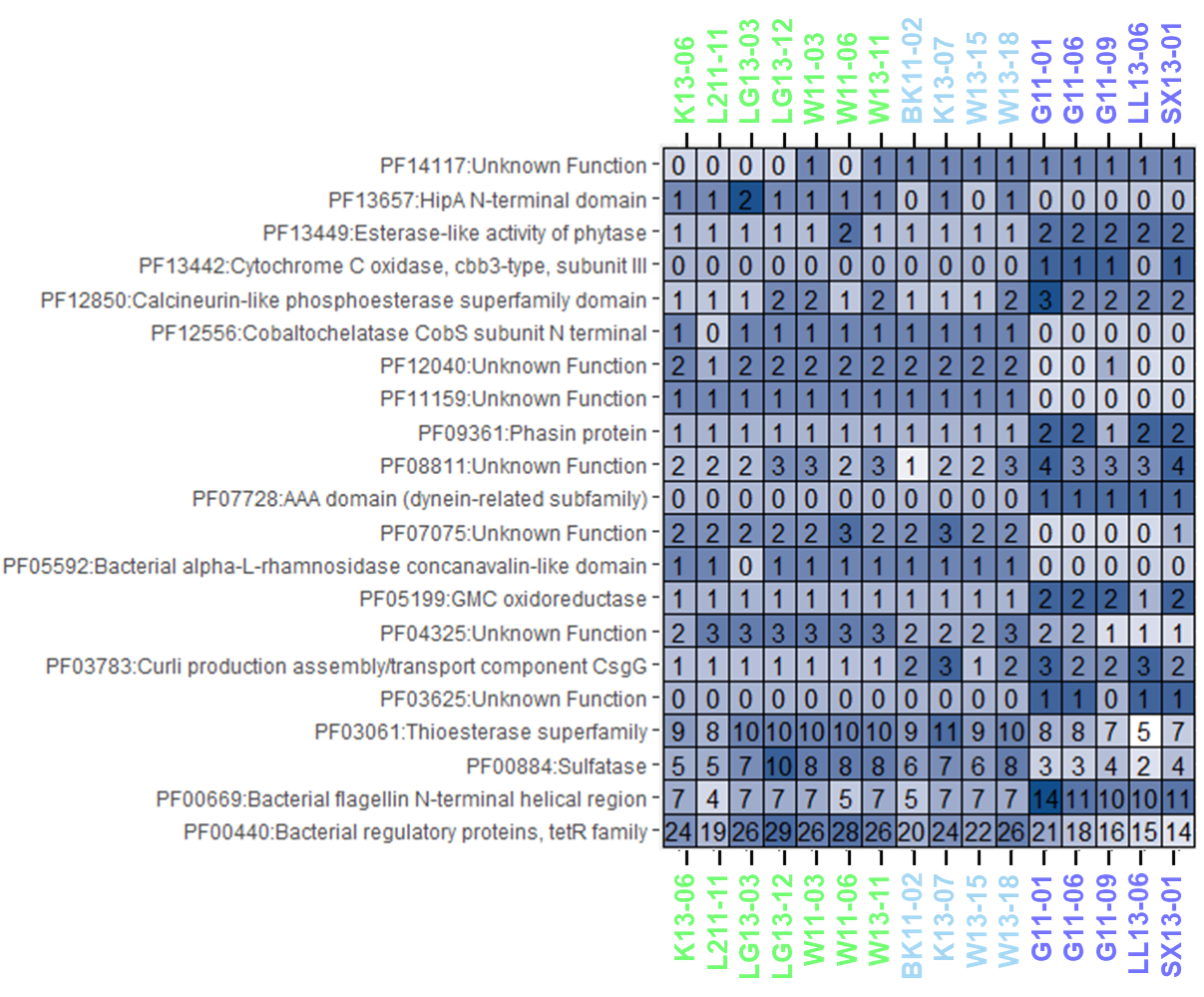


**Table S3.** Genes under positive selection for the low-nutrient branches of heterotrophic bacteria associated with their *Microcystis aeruginosa* host across a phosphorus gradient of freshwater lakes in Michigan. Multiple comparisons corrections are applied to all reported significance values using a false-discovery rate (FDR). All results generated including ω (dN/dS) were computed in the PosiGene software using default parameters.
